# Comparative study of the risk prediction model of early postoperative frailty in elderly enterostomy patients based on machine learning methods

**DOI:** 10.3389/fmed.2024.1404557

**Published:** 2024-07-09

**Authors:** Zhang Ya-juan, Dong Fang-hui, Xue Yi-wei, Lv Gui-fen, Hu San-lian, Ma Li-li

**Affiliations:** ^1^Department of Nursing, Shanghai Sixth People's Hospital Affiliated to Shanghai Jiao Tong University School of Medicine, Shanghai, China; ^2^Department of Nursing, Shanghai East Hospital, School of Medicine, Tongji University, Shanghai, China

**Keywords:** colorectal cancer, enterostomy, frailty, machine learning method, predictive model

## Abstract

**Objective:**

Based on machine learning method, four types of early postoperative frailty risk prediction model of enterostomy patients were constructed to compare the performance of each model and provide the basis for preventing early postoperative frailty of elderly patients with enterostomy.

**Methods:**

The prospective convenience sampling method was conducted and 362 early postoperative enterostomy patients were selected in three hospitals from July 2020 to November 2023 in Shanghai, four different prediction models of Support Vector Machine (SVM), Bayes, XG Boost, and Logistic regression were used and compared the test effects of the four models (MCC, F1, AUC, and Brier index) to judge the classification performance of the four models in the data of this study.

**Results:**

A total of 21 variables were included in this study, and the predictors mainly covered demographic information, stoma-related information, quality of life, anxiety and depression, and frailty. The validated models on the test set are XGBoost, Logistic regression, SVM prediction model, and Bayes on the MCC and F1 scores; on the AUC, XGBoost, Logistic regression, Bayes, and SVM prediction model; on the Brier scores, Bayes, Logistic regression, and XGBoost.

**Conclusion:**

XGBoost based on machine learning method is better than SVM prediction model, Logistic regression model and Bayes in sensitivity and accuracy. Quality of life in the early postoperative period can help guide clinical patients to identify patients at high risk of frailty and reduce the incidence of early postoperative frailty in elderly patients with enterostomy.

## Introduction

The 2023 Global Cancer Report highlights that colorectal cancer remains the third most common cancer worldwide and ranks second in terms of mortality (1). Enterostomy is a crucial treatment for colorectal cancer (2). During the initial postoperative phase, factors such as decreased physical function, alterations in excretion patterns, gastrointestinal issues, and the effects of radiotherapy and chemotherapy can contribute to the development of frailty (3–5). Research indicates that frailty can occur in as many as 92.2% of cases (4).

Frailty is a multidimensional syndrome encompassing independent physiological, psychological, and social factors (6). Research indicates that early postoperative frailty can decrease patient treatment tolerance, raise the likelihood of postoperative complications and mortality, and significantly impact postoperative functional recovery (3, 4). Some studies suggest that colorectal malignancies and associated surgeries induce an “acute stress state”, with the risk of complications and mortality within 30 days being closely linked to the patient's frailty status (7, 8). Early detection of frailty and accurate prediction of its occurrence risk hold significant practical implications for managing frailty.

Machine learning has emerged as a powerful statistical analysis method in recent years, offering the ability to handle high-dimensional variables, non-linear relationships, and complex interactions between variables. This approach allows researchers to explore numerous potential risk factors, select the best features through appropriate algorithms without making prior assumptions, and then build and optimize the model parameters to achieve accurate prediction results (9, 10). Logistic is a classic classification method, which is classified by calculating the probability that samples belong to a certain category. It is simple, intuitive and easy to explain. It can provide the degree of contribution of each independent variable to the predicted results (i.e., weight), and help clinicians and researchers identify the factors most associated with the risk of frailty (11). SVM is a classification method based on statistical learning theory, it is able to find a hyperplane in high dimensional space, separate different categories of samples, and has good generalization ability, SVM has good processing ability for high dimensional data and non-linear problems, can effectively deal with multivariate and complex prediction problems in this study (12). Bayes is a classification method based on the Bayes theorem and the independent assumption of feature conditions. It is simple, efficient, and can achieve good classification effect in some cases (10). Bayes classifier can make full use of the existing prior knowledge and data to make probabilistic prediction, which makes the prediction results more reliable and credible (10). XGBoost is an optimization algorithm based on gradient lifting decision tree. It improves the traditional gradient. Therefore, in this study, we employed four different methods (SVM, Bayes, XG Boost, and Logistic) to develop a risk prediction model for early postoperative frailty in elderly enterostomy patients. By comparing the predictive performance of these models, we aim to provide valuable insights for developing improved risk prediction models for this patient population in the future.

## Methods

### Study population

By convenience sampling, 362 patients who underwent enterostomy surgery in Shanghai from July 2020 to November 2023 and met the inclusion and exclusion criteria were selected as the study subjects of this study. Inclusion criteria: Patients diagnosed with colorectal cancer and enterostomy; aged 60 years; 7 days after surgery; willing to participate in the study, normal cognitive function and communication skills. Exclusion criteria: History of mental illness; Patients clinically diagnosed with other malignancies, severe septic shock, and multiple organ failure. Based on the sample size calculation formula (11), the study subjects should have more than 10 times the number of weak events compared to the prediction index. For the 23 candidate risk factors for this study, at least 230 elderly enterostomy patients should be included. According to the previous investigation of the research group, the incidence of early postoperative frailty in elderly colorectal cancer patients was 92.2% (4), namely 230/92.2% = 241. According to the rate of 10% follow-up, at least 267 patients need to be investigated, and a total of 362 patients were included in the study period. This study received approval from the hospital ethics committee [Batch No.: [2022] (001)], and all patients provided informed consent by signing the necessary documents.

#### Related definition criteria of frailty risk predictors and outcome

The research group conducted a literature review to identify risk factors for early weakness following enterostomy in elderly patients. A systematic literature search was conducted using key terms such as “ostomy,” “enterostomy,” “colostomy,” “ileostomy,” “intestinal stoma,” “stoma,” “weakness,” “weak,” “frail,” “frailty,” and “complications.” Various databases including CBM, WanFang Data, CNKI, VIP, Embase, the Cochrane Library, Web of Science, ScienceDirect, and PubMed were searched for relevant studies. Two investigators with expertise in evidence-based research conducted the literature screening, resulting in the retrieval of 1,419 documents. After screening, 13 articles met the inclusion criteria. Quality assessment of the included studies was independently performed by the investigators using the Newcastle-Ottawa Scale for case-control and cohort studies, and the Agency for Healthcare Research and Quality criteria for cross-sectional studies. Two investigators extracted risk factors from the literature independently. Two rounds of Delphi expert consultations were conducted to assess the clinical relevance of these risk factors. The selection criteria for the experts are the ostomy specialist nurses who have worked for 10 years, and 10 ostomy specialist nurses participated. The research group discussed the formation of a questionnaire of risk factors for early frailty after elderly enterostomy. Subsequently, a questionnaire was developed to evaluate early frailty risk factors post-enterostomy in the elderly population. These factors include gender (4), age (13) education (4), marital status (14), acute and chronic diseases (15), self-perceived health status (14) monthly income (4), stoma complications (such as fecal skin dermatitis, stoma bleeding, injury, allergic dermatitis, necrosis, and mucosal separation) (16), receipt of chemoradiotherapy (17), stoma acceptance (16), sleep quality (18), social support (4), anxiety (14), preoperative frailty (19), and cancer stage (16). The recovery process post-enterostomy was divided into three stages: early surgery, recovery period, and post-discharge recovery. Early surgery refers to the period up to 1 month post-operation, while the recovery period extends beyond discharge. Patients included in the study had undergone enterostomy within 1 week of surgery (4). Using the Tilburg Frailty Scale (20), patients were assessed across three dimensions: physical, psychological, and social, with a total score range of 0–15 and a score of five indicating frailty. Higher scores corresponded to greater frailty severity.

#### Model building and validation

In this study, data was analyzed using a machine learning algorithm. A total of 362 samples were randomly divided into a training set and a validation set in a 7:3 ratio through the random grouping function in Python (train test split). The training set, consisting of 70% (253 cases), was used for model building, while the remaining 30% (109 cases) served as the test set for model evaluation. Given the small sample size and the dichotomous nature of the predicted outcome, XG Boost, Logistic Regression, SVM, and Bayes, these 4 models were chosen for analysis. The performance of each model was assessed based on metrics such as accuracy, precision, recall rate (sensitivity), specificity, F1 score, area under the ROC curve, and Brier score.

#### Quality control

The personnel involved in the survey and database construction have received unified training; the survey is arranged on the 7th day after the operation; double input and third party error checking to ensure the accuracy of the data. To ensure the consistency of the four models, a statistical expert was invited to handle the operation. The research team includes clinical medical experts, clinical nursing experts, ostomy specialist nurses, nursing undergraduates, and statistical experts.

#### Statistical method

In this study, the data were statistical analyzed by SPSS 23.0 software (IBM, Armonk, NY, USA), and the statistical analysis was completed by Python 3.11.0 software. Measurement data meeting the normal distribution were expressed as mean ± standard deviation ($\bar{\text{x}}\;\pm$ s) and *t*-test was used for comparison between groups. Measurement data with non-normal distribution are presented as median, quartile [M (Q1, Q3)], and comparisons between groups were performed using the Mann-Whitney *U*-test. Count data were expressed as frequency and composition ratio (%), and χ^2^-test was used for comparison between groups. The performance of the four models was evaluated by accuracy, precision, recall (sensitivity), specificity, F1 score, area under the ROC curve, and brief score, and *P* < 0.05 was considered as a statistically significant difference.

## Results

### General characteristics of the 362 patients with enterostomy

Among the 362 elderly patients with enterostomy, there were 187 males and 175 females. In terms of age distribution, 189 patients were 65–69 years old, 44 were below junior high school level, 116 had completed high school or technical secondary school, 56 had attended college, and 14 had completed undergraduate studies or above. Additionally, 184 patients were accompanied by family members, while 47 were accompanied by nannies or escorts. The general condition of the patients and the detection rate are detailed in Table 1.

**Table 1:** General data and analysis of frailty-related risk predictors in 362 elderly patients with enterstomy.

**Variable**	**Category**	**Non-frailty (%)**	**Frailty (%)**	***t*/*Z*/Chi-square value**	***p***
Gender	Male	61 (78.20%)	233 (82.00%)	0.591	0.442
	Female	17 (21.80%)	51 (18.00%)		
Age	60–69 years old	75 (96.20%)	224 (78.90%)	12.712	<0.001
	≥70 years old	3 (3.80%)	60 (21.10%)		
Educational background	Junior high school and below	6 (7.70%)	69 (24.30%)	66.063	<0.001
	High school and technical secondary school	28 (35.90%)	156 (54.90%)		
	Junior college	27 (34.60%)	56 (19.70%)		
	Undergraduate course	17 (21.80%)	3 (1.10%)		
Marital status	Having a spouse (including remarriage)	73 (93.60%)	174 (61.30%)	29.494	<0.001
	No spouse (including divorce, widowed, unmarried, etc.)	5 (6.40%)	110 (38.70%)		
Nationality	Han nationality	53 (67.90%)	180 (63.40%)	0.557	0.456
	Others	25 (32.10%)	104 (36.60%)		
Religious belief	Yes	61 (78.20%)	228 (80.30%)	0.164	0.686
	No	17 (21.80%)	56 (19.70%)		
Working status	Employed	12 (15.40%)	14 (4.90%)	10.034	0.002
	Unemployed	66 (84.60%)	270 (95.10%)		
Self-conscious health status	Very bad	4 (5.10%)	27 (9.50%)	84.607	<0.001
	Bad	22 (28.20%)	197 (69.40%)		
	Common	35 (44.90%)	58 (20.40%)		
	Good	17 (21.80%)	2 (0.70%)		
Acute diseases and/or chronic diseases	No	66 (84.60%)	192 (67.60%)	10.583	0.005
	One disease	11 (14.10%)	61 (21.50%)		
	Two or more diseases	1 (1.30%)	31 (10.90%)		
Family monthly income	<2,000	0 (0.00%)	7 (2.50%)	113.015	<0.001
	2,000–2,999	8 (10.30%)	116 (40.80%)		
	3,000–3,999	17 (21.80%)	129 (45.40%)		
	4,000–4,999	31 (39.70%)	23 (8.10%)		
	5,000–5,999	20 (25.60%)	8 (2.80%)		
	≥7,000	2 (2.60%)	1 (0.40%)		
Mode of payment	Self-paid	6 (7.70%)	10 (3.50%)	4.764	0.092
	Residents' medical insurance	68 (87.20%)	268 (94.40%)		
	Commercial insurance	4 (5.10%)	6 (2.10%)		
Expenditure pressure	No	39 (50.00%)	145 (51.10%)	0.027	0.869
	Yes	39 (50.00%)	139 (48.90%)		
Stoma complications	No	72 (92.30%)	189 (66.50%)	20.184	<0.001
	Yes	6 (7.70%)	95 (33.50%)		
Care giver	Family members	61 (78.20%)	224 (78.90%)	0.016	0.898
	House maid	17 (21.80%)	60 (21.10%)		
Activity times	Never	6 (7.70%)	13 (4.60%)	5.769	0.123
	1–3 times per day	36 (46.20%)	142 (50.00%)		
	4–6 times per day	33 (42.30%)	127 (44.70%)		
	>6 times per day	3 (3.80%)	2 (0.70%)		
Type of stoma	Ileum	36 (46.20%)	148 (52.10%)	0.869	0.351
	Colon	42 (53.80%)	136 (47.90%)		
Intraoperative chemotherapy	No	75 (96.20%)	235 (82.70%)	8.942	0.003
	Yes	3 (3.80%)	49 (17.30%)		
Sleeping		9 (7, 10)	12 (10, 13)	−8.232	<0.001
Life of quality		6 (5, 6)	5 (5, 5)	−9.913	<0.001
Anxiety		43 (38.75, 46)	48 (45, 50)	−9.747	<0.001
Social support		27 (23, 32)	26 (22, 29.75)	−2.375	0.018

### Characteristics of risk factors of the model

**Figure 1:**
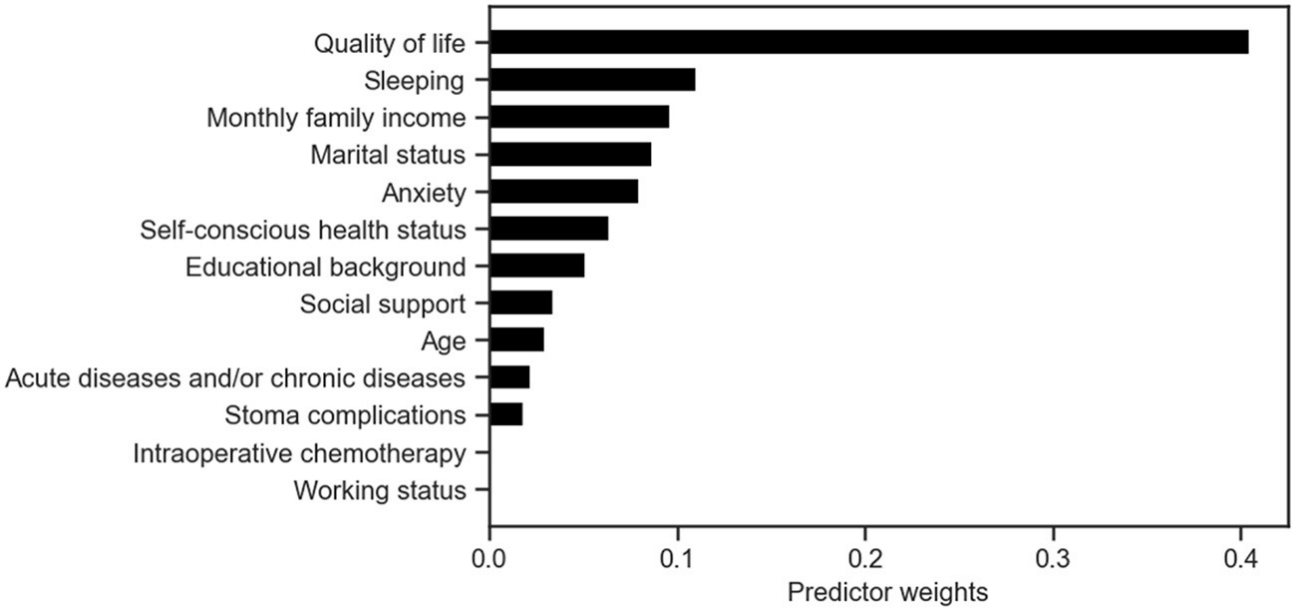
Predictor weights in the XGBoost model for early postoperative frailty risk prediction in elderly enterostomy patients.

**Figure 2:**
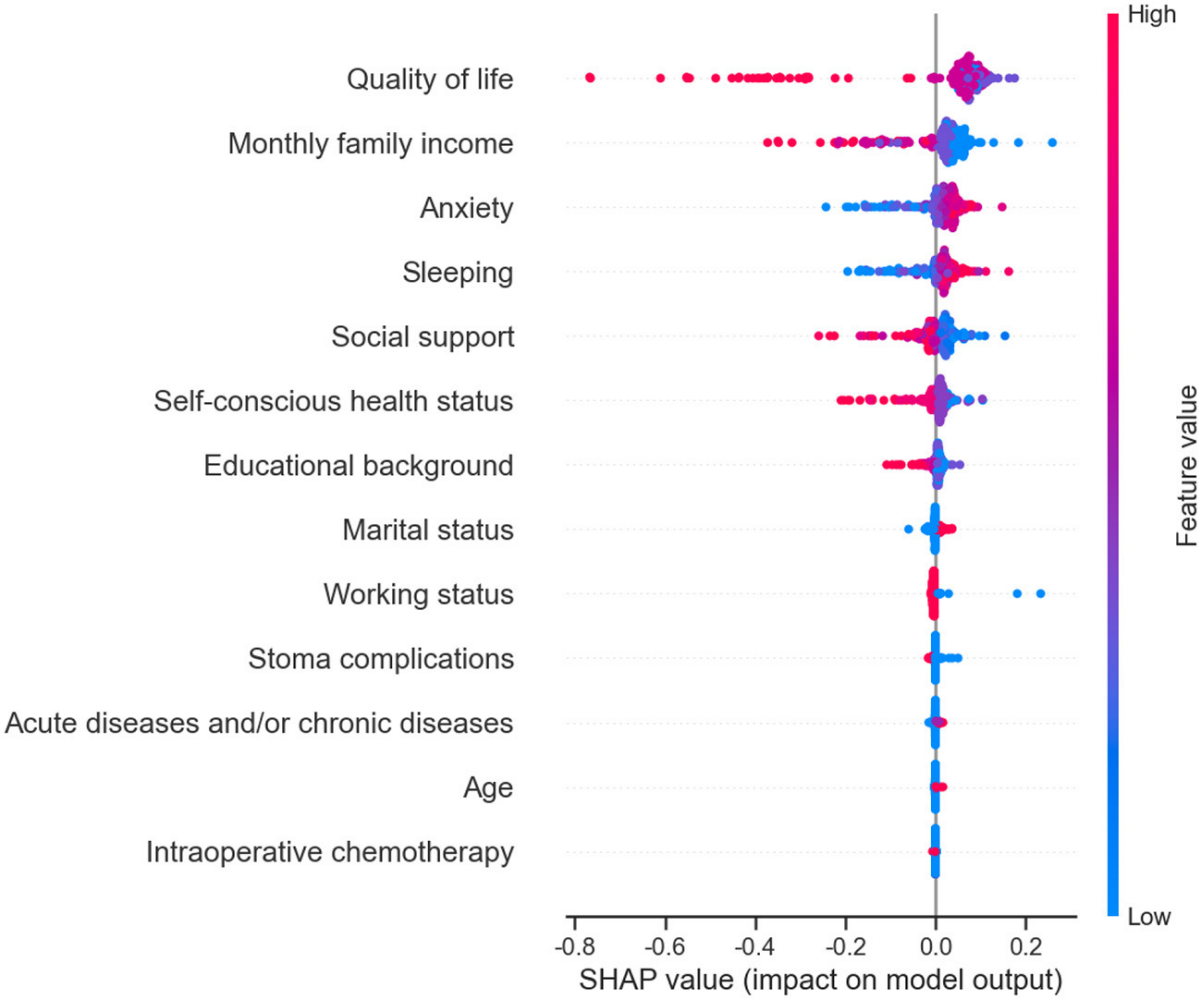
Predictor SHAP values in the early postoperative frailty SVM model in elderly patients with enterostomy.

The XG Boost prediction model identified quality of life, sleep, monthly family income, marital status, anxiety, and conscious health status as the top six factors influencing the model. Similarly, the SHAP values in the SVM prediction model revealed that these same six indicators had the most significant impact on the model. Additionally, the Logistic regression prediction model considered age, work status, conscious health status, sleep, quality of life, anxiety, and social support as the seven key predictors. More detailed information can be found in Table 2 and Figures 1, 2.

**Table 2:** Frailty-related risk predictors of elderly patients with enterstomy based on logistic regression model.

**Variable**	**Regression coefficient**	**Standard error**	**Wald**	***P***	**OR**	**95% CI**
Constant	1.145	4.707	0.059	0.808	3.144	
Age	3.184	1.358	5.494	0.019	24.139	1.685–345.875
Working status	−3.266	1.102	8.776	0.003	0.038	0.004–0.331
Self-conscious healthy health (reference: poor)			21.963	<0.001		
Bad	−2.171	1.149	3.566	0.059	0.114	0.012–1.086
Common	−4.953	1.337	13.728	<0.001	0.007	0.001–0.097
Good	−3.679	2.271	2.624	0.105	0.025	0.001–2.164
Sleeping	0.687	0.137	24.995	<0.001	1.987	1.518–2.601
Life of quality	−4.842	1.098	19.460	<0.001	0.008	0.001–0.068
Anxiety	0.642	0.118	29.793	<0.001	1.900	1.509–2.392
Social support	−0.199	0.059	11.204	<0.001	0.820	0.729–0.921

### Comparison between the performance of the four models

**Figure 3:**
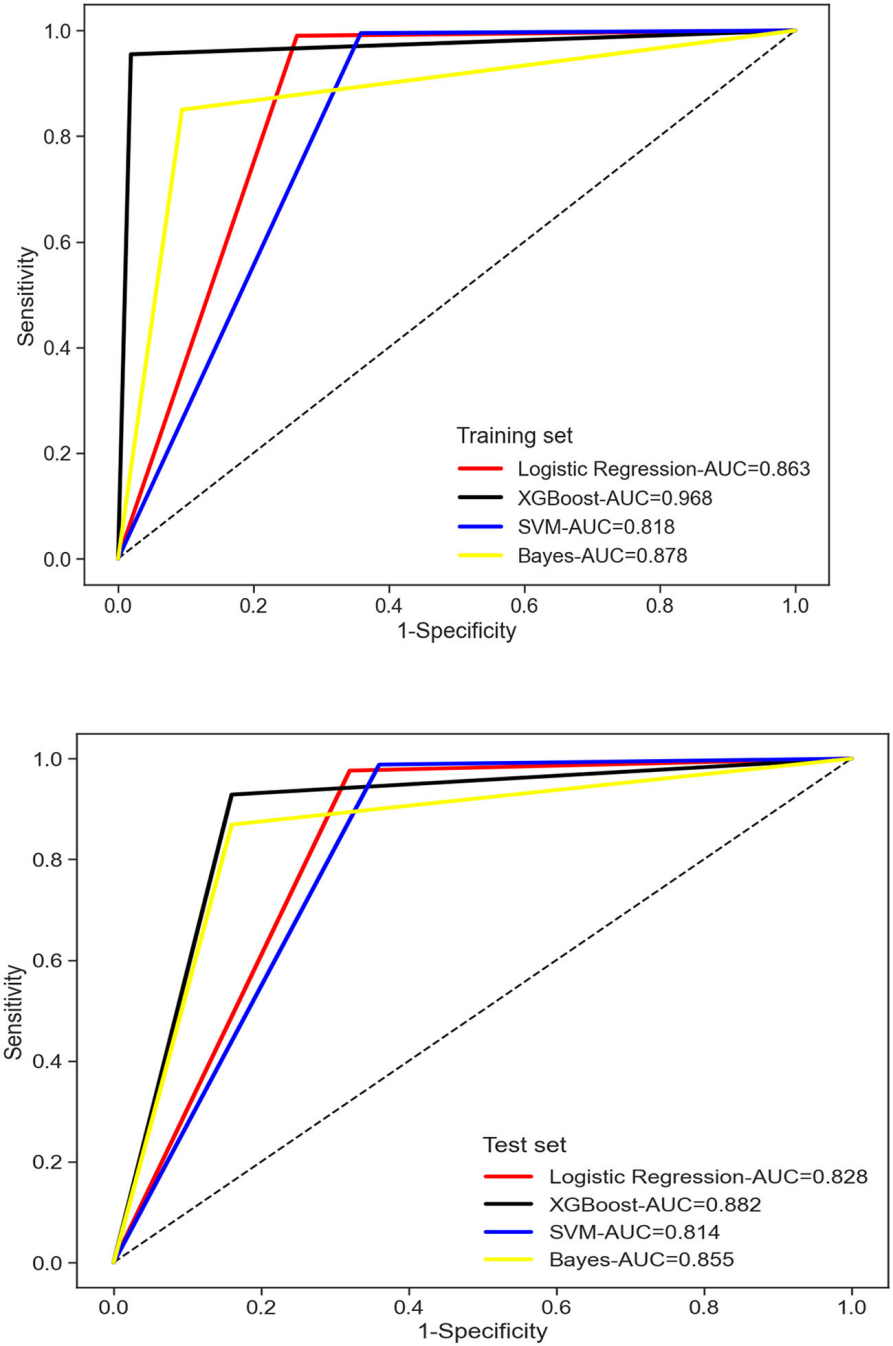
Comparison amongst plot of area under the curve in the early postoperative frailty risk model for four elderly enterostomy patients.

Among the four models, XG Boost achieved the highest MCC value in both the training and test sets. In the training set, XG Boost also had the highest F1 value, while in the test set, SVM and Logistic had the highest F1 values. The Brier scores for prediction models in the training set were 0.063 for Logistic, 0.040 for XG Boost, 0.079 for SVM, and 0.138 for Bayes. In the test set, the Brier scores were 0.092 for Logistic, 0.092 for XG Boost, 0.092 for SVM, and 0.137 for Bayes. Internal verification results on the model test set and training set indicate that the sensitivity and calibration of the XGBoost model are superior to the other three models. For more detailed information, refer to Table 3 and Figure 3.

**Table 3:** Comparison results of the four types models.

**Data set**	**Model**	**MCC**	**F1**	**AUC**	**Brier**
Training set	Logistic	0.802	0.961	0.863	0.063
	XGBoost	0.891	0.974	0.968	0.040
	SVM	0.750	0.952	0.818	0.079
	Bayes	0.666	0.907	0.878	0.138
Test set	Logistic	0.727	0.943	0.828	0.092
	XGBoost	0.749	0.940	0.882	0.092
	SVM	0.728	0.943	0.814	0.092
	Bayes	0.655	0.907	0.855	0.137

## Discussion

Frailty is characterized by decreased body resistance and increased vulnerability, manifesting in various physiological, psychological, and social aspects. Frailty is a dynamic and reversible process. Identifying key risk factors and implementing proactive measures based on these factors can decelerate the progression of weakness in patients and enhance their frailty condition. Currently, some researchers are utilizing machine learning techniques to investigate relevant risk factors for frailty in elderly enterostomy patients and develop predictive models to diagnose or anticipate frailty at an early stage. This endeavor holds significant value in averting frailty onset and enhancing patient quality of life. Notably, the occurrence of early postoperative frailty in elderly colorectal cancer patients was notably high at 78.4%, surpassing the findings of ELABBAS et al. study (50%) (15) and falling below the previous research group investigation (92.2%) (4). The higher incidence of frailty in this study may be attributed to the inclusion of elderly patients over 65 years old, as age is a significant risk factor for frailty. Moreover, older elderly patients often lack knowledge and skills for stoma self-care, making them more susceptible to weakness. The research group conducted preliminary investigations on patients within 7 days post-surgery, while this study focused on patients on the 7th day after surgery. It is possible that cancer itself contributes to a wasting disease. Additionally, elderly patients experience a heightened stress response post-surgery, leading to a more significant decrease in activity levels. Identifying frailty risk factors is crucial for frailty assessment. The research group developed the initial questionnaire based on literature evidence and preliminary findings, refining its clinical applicability through two rounds of expert consultations. Subsequently, the research group finalized the questionnaire on early frailty risk factors following enterostomy in the elderly.

Effective management of frailty relies on the prevention of various risk factors. The results of the univariate analysis revealed that the risk factors for elderly enterostomy patients include age, educational background, marital status, self-reported health status, monthly family income, ostomy complications, sleep quality, anxiety levels, social support, presence of acute and chronic diseases, intraoperative chemotherapy, and employment status. Previous literature has highlighted a scarcity of studies focusing on the risk factors associated with enterostomy frailty. For instance, Chen et al. (11) developed a risk prediction model for stroke patients, identifying risk factors such as living alone, age, physical activity, smoking habits, diabetes, hypertension, sleep disturbances, history of falls, and daily living abilities. The results of age, acute and chronic diseases, and sleep quality are consistent with previous findings. A longitudinal health life survey (CLHLS) was conducted to develop a risk prediction model for frailty in elderly individuals across 23 provinces in China (21). The risk prediction factors, including age, family income, social support, and marital status, align with the results of this study. This may be attributed to differences in the study population and varying risk factors. The analysis using XG Boost and SVM models revealed that quality of life emerges as the primary risk factor for frailty. The findings suggest that a lower quality of life is associated with higher levels of frailty, corroborating previous research by CROCKERT (22). Elderly patients with frail ostomy tend to have lower quality of life, emphasizing the importance of early postoperative evaluation, timely screening, and interventions to prevent and manage frailty, thereby enhancing overall quality of life. Furthermore, the XG Boost model analysis identified sleep quality as a secondary risk factor for frailty. The review did not find any reported risk factors related to frailty, but researchers explored the relationship between frailty and sleep quality (23). The results indicate that elderly patients with frailty experience poorer sleep quality, which may serve as a precursor to significant emergency stimuli. The formation of a stoma can have a profound impact on the physiological, psychological, and social aspects of patients, leading to sleep disorders. Poor sleep quality is often associated with anxiety, depression, fatigue, gait instability, decreased activity levels, and increased frailty.

In this study, Support Vector Machine (SVM), Bayes, XG Boost, and Logistic Regression were employed to develop a predictive model for early and postoperative weakness in elderly colorectal cancer patients. The XG Boost model outperformed the other models in terms of area under the curve, specificity, Matthews Correlation Coefficient (MCC), and F1 score. Additionally, the XG Boost model exhibited higher sensitivity and calibration, making it the most effective model (24). XG Boost is a Boosting integrated learning machine algorithm capable of addressing both classification and regression problems.

The combination of multiple weak learners can lead to a strong learner. XG Boost is a powerful method for estimating AGB, effectively mitigating model overfitting and enhancing prediction accuracy. The risk factors, ranked from high to low, include quality of life, sleep, monthly family income, marital status, anxiety, self-perceived health status, highest education level, social support, age, current acute and chronic diseases, stoma complications, intraoperative chemotherapy, and work status. When assessing the results, AUC is considered the primary indicator (25). The AUC values for the four models are as follows: SVM 0.818, Bayes 0.878, XG Boost 0.968, and Logistic regression 0.863, indicating that XG Boost > Bayes ≈ Logistic regression > SVM. Some researchers emphasize that calibration is crucial in evaluating model performance, as it reflects the accuracy of risk estimation (26). All four models showed a calibration degree much <0.25, suggesting that they all provide reliable predicted outcomes. The study demonstrates that XGBoost outperforms other models in providing doctors with accurate risk assessment results. This enables doctors to tailor personalized treatment plans and care plans based on the patient's risk assessment, ultimately enhancing treatment outcomes and improving patients' quality of life.

## Conclusion

In conclusion, this study applied SVM, Bayes, XG Boost and Logistic to build the risk prediction model for elderly colorectal cancer patients. By analyzing the performance of MCC, F1, AUC and Brier scores, this study shows that the XG Boost algorithm was optimal, Bayes is similar to the traditional Logistic regression algorithm, and the SVM algorithm was the worst. This study also provides relevant indicators of early frailty predictors and risk factors after elderly enterostomy, which can help clinical nurses to conduct more accurate assessment, guide the prevention and treatment of frailty, and improve the quality of life of patients. However, the sample size of this study was small with only 362 cases, and external validation of the model can be conducted by further expanding the sample size in the future.

## Limitation

This study acknowledges certain limitations: although 362 patients were included, the sample size may not fully capture the overall risk of frailty in older bowel stoma patients; the comparison of four prediction models without exploring all possible algorithms may overlook models with greater prediction accuracy. To address these limitations, future research should focus on expanding sample size, enhancing data diversity, testing additional machine learning algorithms, fine-tuning model parameters, improving prediction accuracy, increasing model interpretability, and validating performance on multiple external datasets.

## Data availability statement

The original contributions presented in the study are included in the article/supplementary material, further inquiries can be directed to the corresponding authors.

## Ethics statement

The studies involving humans were approved by the Ethics Committee of Shanghai East Hospital. The studies were conducted in accordance with the local legislation and institutional requirements. The participants provided their written informed consent to participate in this study.

## Author contributions

ZY-j: Writing – original draft, Writing – review & editing. DF-h: Conceptualization, Methodology, Writing – review & editing. XY-w: Data curation, Investigation, Methodology, Writing – original draft. LG-f: Data curation, Formal analysis, Investigation, Writing – review & editing. HS-l: Methodology, Supervision, Writing – review & editing. ML-l: Writing – original draft, Writing – review & editing.
